# Integrative utility of long read sequencing-based whole genome analysis and phenotypic assay on differentiating isoniazid-resistant signature of *Mycobacterium tuberculosis*

**DOI:** 10.1186/s12929-021-00783-x

**Published:** 2021-12-18

**Authors:** Ming-Chih Yu, Ching-Sheng Hung, Chun-Kai Huang, Cheng-Hui Wang, Yu-Chih Liang, Jung-Chun Lin

**Affiliations:** 1 Division of Pulmonary Medicine, Department of Internal Medicine, Wan Fang Hospital, Taipei Medical University, Taipei, Taiwan; 2 School of Respiratory Therapy, College of Medicine, Taipei Medical University, Taipei, Taiwan; 3 Pulmonary Research Center, Wan Fang Hospital, Taipei Medical University, Taipei, Taiwan; 4 Ph.D. Program in Medical Biotechnology, College of Medical Science and Technology, Taipei Medical University, Taipei, Taiwan; 5 Department of Laboratory Medicine, Wan Fang Hospital, Taipei Medical University, Taipei, Taiwan; 6 School of Medical Laboratory Science and Biotechnology, College of Medical Science and Technology, Taipei Medical University, 250 Wu-Hsing Street, Taipei, 11031 Taiwan

**Keywords:** InhA, Isoniazid, KatG, MinION, *Mycobacterium tuberculosis*

## Abstract

**Background:**

With the advancement of next generation sequencing technologies (NGS), whole-genome sequencing (WGS) has been deployed to a wide range of clinical scenarios. Rapid and accurate classification of drug-resistant *Mycobacterium tuberculosis* (*MTB*) would be advantageous in reducing the amplification of additional drug resistance and disease transmission.

**Methods:**

In this study, a long-read sequencing approach was subjected to the whole-genome sequencing of clinical MTB clones with susceptibility test profiles, including isoniazid (INH) susceptible clones (*n* = 10) and INH resistant clones (*n* = 42) isolated from clinical specimens. Non-synonymous variants within the *katG* or *inhA* gene associated with INH resistance was identified using Nanopore sequencing coupled with a corresponding analytical workflow.

**Results:**

In total, 54 nucleotide variants within the *katG* gene and 39 variants within the *inhA* gene associated with INH resistance were identified. Consistency among the results of genotypic profiles, susceptibility test, and minimal inhibitory concentration, the high-INH resistance signature was estimated using the area under the receiver operating characteristic curve with the existence of Ser315Thr (AUC = 0.822) or Thr579Asn (AUC = 0.875).

**Conclusions:**

Taken together, we curated lists of coding variants associated with differential INH resistance using Nanopore sequencing, which may constitute an emerging platform for rapid and accurate identification of drug-resistant MTB clones.

## Introduction

Infection with *Mycobacterium tuberculosis* (MTB) is still considered a health threat with about 10 million incident cases of tuberculosis (TB) and 1 million deaths every year [1]. Efforts of epidemiological control of TB are hampered by increased drug-resistant MTB strains and the absence of rapid and accurate assays for classifying drug resistance profiles. Among the 10 million TB cases, about 19% of newly treated cases or 43% of previously treated cases are resistant to at least one frontline antibiotic [1]. Furthermore, isoniazid (INH) resistance is the most common monoresistance, and it is associated with treatment failure and progression to multidrug-resistant TB [1]. Catalase-peroxidase encoded by the *katG* gene converts INH to its active form, which in turn acts by blocking the biosynthesis of mycolic acid which is required for cell wall synthesis [2]. Active INH is next targeted by enoyl acyl carrier protein reductase (inhA) and beta-ketoacyl ACP synthase (kasA) [2]. INH resistance is therefore exclusively associated with mutations in the katG or inhA gene [3].

Drug resistance in MTB is routinely evaluated using phenotypic methods, such as susceptibility tests or genotypic approaches [4]; however, discordance between distinct assays was frequently noted [5]. With advancement of high-throughput sequencing, the abovementioned discordance was evaluated using results of whole-genome sequencing (WGS) [6]. Nevertheless, the efficiency and accuracy of amplicon-based analyses toward the MTB genome encounter interference with the high-GC region [7]. Herein, the Oxford Nanopore Technologies (ONT) long-read sequencing platform and corresponding analytic workflow were subjected to the WGS of susceptible, low INH-resistance, and high INH-resistance MTB. Correlations of susceptible test results and minimal inhibitory concentration (MIC) tests with mutant profiles of the katG and inhA genes were monitored. Taken together, the ONT sequencing platform can potentially serve as an auxiliary test toward a precise diagnosis and treatment of MTB disease.

## Materials and methods

### Study overview

We conducted WGS to identify non-synonymous variants within the katG and inhA genes in three groups of MTB clones, including (1) susceptible isolates, (2) low INH-resistant isolates, and (3) high INH-resistant isolates.

### Ethics statement of the study cohort and sample collection

Enrollment of clinical isolates was reviewed and approved by the Institutional Review Board of Taipei Medical University (approval no. N201912076). In total, 50 MTB isolates were collected from clinical specimens at Taipei Municipal Wan Fang Hospital.

### Susceptible test

All isolates in this study were examined at the Department of Laboratory Medicine, Taipei Municipal Wan Fang Hospital using an agar proportion assay in accordance to the Clinical and Laboratory Standards Institute (CLSI) methods. In brief, bacterial suspensions with a turbidity equivalent to 1.0 McFarland standard were prepared from fresh MTB colonies cultured on Lowenstein-Jensen medium. After examination of the turbidity using a nephelometer, the suspension was applied as the inoculum for all dilutions. One hundred microliters of 10^−2^ and 10^−4^ dilutions of the standard inoculum was spread on 7H10 agar with or without an anti-TB drugs, including Isoniazid (INH), Rifampin (RIF), Ethambutol (EM), Streptomycin (S) and concentrations in microgram/milliliter. Drug resistance was defined as more than 1% colony growth in the presence of the drug compared to colony growth in the absence of the drug.

### Extraction of high-molecular weight (HMW) genomic DNA

MTB isolates were cultured on Lowenstein-Jensen media in the absence of an anti-MTB drug. Multiple colonies from a single isolate were emulsified and inactivated in 200 μL nuclease-free water at 95 °C for 15 min. Genomic (g)DNA was extracted using the Presto™ Mini gDNA Bacteria Kit (Geneaid, Taipei, Taiwan) according to the manufacturer’s instructions. The DNA concentration was measured using a fluorometric kit (GeneCopoeia, Rockville, MD, USA) and a Qubit fluorometer (ThermoFisher Scientific, Wilmington, DE, USA).

### Whole genome sequencing and variant identification

WGS of MTB gDNA was conducted using a third-generation long read-sequencing approach. In brief, 500 ng of extracted gDNA was first homogenized with 8000 bps using a g-TUBE device (Covaris, Woburn, MA, USA) according to the manufacturer’s instructions. The fractionated gDNA was subjected to library construction using a Ligation Sequencing Kit (SQK-LSK109; Oxford Nanopore Technologies (ONT), Oxford, UK) coupled with a Native Barcoding Expansion kit (EXP-NBD104 and 114; ONT) according the manufacturer’s protocol. The barcoded library was captured, washed, and eluted from magnetic beads (AMPure XP, Beckman Coulter, High Wycombe, UK). Seven hundred nanograms of the pooled library was loaded and sequenced on MinION flow cells (FLO-MIN106D R9.4.1; MinION instrument; ONT). The sequence read number of each sample was 160,000 ~ 200,000 to meet a reading depth of 200. For analysis of sequencing results, the MinION-sequenced reads were uploaded through the EPI2ME desktop agent (ONT), and the quality and quantity of sequencing results were assessed using the EPI2ME website algorithm (https://epi2me.nanoporetech.com). The coverage rate of sequenced reads to the MTB reference was estimated by applying FastQ custom alignment (ONT). Analytical results of variant classification were aligned to the MTB reference (*M. tuberculosis* H37Rv, NC_000962.3) using Bacterial Small Variant Calling workflow composed of the Medaka variant calling pipeline with long-read datasets via the EPI2ME Labs Launcher (ONT). The sequencing quality, reading depth, and variant calling with long-read datasets were repeatedly assessed using the CLC genomics workbench (Qiagen v21.0.3; CLC bio, Denmark).

### Minimal inhibitory concentration (MIC) test

Tests were conducted using Sensititre MYCOTB MIC plates (ThermoFisher Scientific) according to the manufacturer’s instructions. In brief, an MTB isolate was subcultured on 7H10 agar (Becton, Dickinson and Co., Sparks, MD, USA). Fresh colonies were resuspended with glass beads in a saline-Tween solution, and the turbidity was adjusted to a McFarland standard of 0.5. After 15 min, 100 µl of the resuspension was poured and mixed with 11 ml 7H9 broth containing oleic acid albumin-dextrose-catalase (Trek Diagnostic Systems, Cleveland, OH). Diluent (100 µl) was inoculated into each well of the MYCOTB plate that was covered with permanent plastic seals and incubated at 37 °C in 5% CO_2_. Plates were monitored on days 7, 10, 14, and 21 using a mirrored viewer. No visible growth with the lowest concentration was considered the MIC of each antibiotic.

### Statistical analysis

Experimental results were statistically analyzed using a one- or two-way analysis of variance (ANOVA) followed by Tukey’s multiple-comparison post-hoc test. Analytical results are presented as the mean ± standard error of the mean (SEM) and considered significant at *p* values of < 0.05 (*p < 0.05; **p < 0.01; ***p < 0.005). The utility of identified variants for predicting INH resistance was evaluated using the receiver operating characteristic (ROC) curve and area under the ROC curve (AUC) ratio as implemented in *R* programming.

## Results

### Phenotypic resistance patterns of clinical MTB isolates

Drug-resistance patterns determined by the agar proportion method are displayed in Table 1. Ten isolates showed susceptibility to INH, rifampin (RIF), ethambutol (EM), and streptomycin (S). Among the 42 drug-resistant clones, 14 isolates were INH-resistant, and other isolates were multidrug-resistant (MDR)-TB (Table 1).

**Table 1:** Results of the drug susceptibility test of MTB isolates enrolled in this study

No.	INH 0.2(μg/mL)	INH 1.0 (μg/mL)	RIF 1.0 (μg/mL)	EM 5.0 (μg/mL)	EM 10.0 (μg/mL)	S 2.0 (μg/mL)	S 10.0 (μg/mL)
1	R	S	S	S	S	S	S
2	R	S	S	S	S	S	S
3	R	S	S	R	S	S	S
4	R	S	R	R	S	R	R
5	R	S	R	R	S	S	S
6	R	S	S	R	S	S	S
7	R	S	S	S	S	S	S
8	R	S	S	S	S	S	S
9	R	S	S	S	S	S	S
10	R	S	S	S	S	R	R
11	R	S	R	S	S	S	S
12	R	S	S	S	S	S	S
13	R	S	R	S	S	S	S
14	R	S	S	S	S	S	S
15	R	S	R	S	S	R	R
16	R	S	R	R	S	S	S
17	R	S	R	S	S	S	S
18	R	S	S	S	S	S	S
19	R	S	S	S	S	S	S
20	R	S	S	S	S	S	S
21	R	R	S	S	S	S	S
22	R	R	R	R	S	R	S
23	R	R	S	S	S	R	S
24	R	R	S	S	S	R	R
25	R	R	S	S	S	R	R
26	R	R	R	R	S	R	R
27	R	R	S	S	S	R	R
28	R	R	S	S	S	S	S
29	R	R	R	S	S	S	S
30	R	R	S	R	S	S	S
31	R	R	S	S	S	S	S
32	R	R	S	S	S	S	S
33	R	R	R	R	S	R	R
34	R	R	S	S	S	R	S
35	R	R	S	S	S	R	S
36	R	R	S	S	S	R	S
37	R	R	S	S	S	S	S
38	R	R	S	R	S	S	S
39	R	R	S	S	S	R	S
40	R	R	S	S	S	R	R
41	R	R	S	R	S	S	S
42	R	R	R	R	S	R	S

### Statistical analysis of long-read sequencing results

**Fig. 1:**
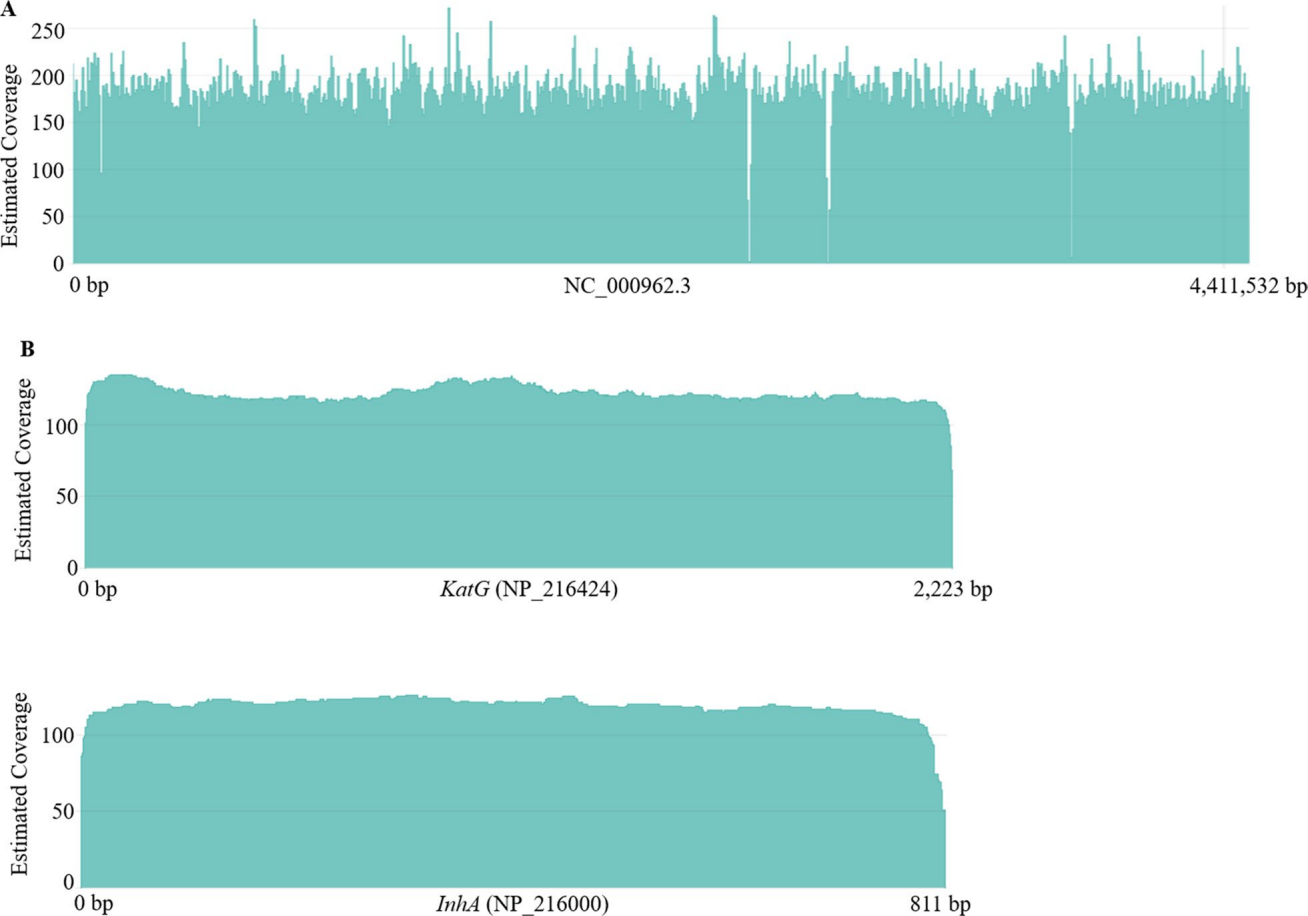
The diagram presents the coverage rate of long-read sequencing reads to the (**A**) whole MTB genome; (**B**) *katG* and *inhA* gene

The HMW gDNA extracted from the clinical isolates was subjected to sequencing using a long-read sequencer (MinION, ONT). Numbers with an average of sequenced and aligned reads per sample were filtered and analyzed using the CLC Genomics Workbench (v.21.0.2, Aarhus, Denmark) and EPI2ME desktop agent algorithm (ONT). As shown in Table 2, no statistical differences in sequencing or alignment efficiencies were identified among all groups. On average, results of the custom alignment with sequencing results using the EPI2ME desktop agent algorithm (ONT) showed more than 95% with over 200 × coverage in all samples enrolled in this study, and sequenced reads could be considered to virtually cover the entire MTB genome (Fig. 1A). Nevertheless, low coverage for a highly-repeated or homopolymeric region within the entire MTB genome was noted in this study.

**Table 2:** Summary statistics of long-read sequencing results in each group

Group	Susceptible isolates (n = 10)	Low isoniazid-resistance (n = 20)	High isoniazid-resistance (n = 22)	*p* value
Number of raw reads (mean; (SD))	382,196 (± 17,321)	333,823 (± 15,702)	356,660 (± 14,407)	> 0.1
Number of aligned reads (mean; (SD))	366,547 (± 10,121)	322,075 (± 8,332)	339,176 (± 11,540)	> 0.1
Correctly classified (% (SD))	95.91 (5.64)	96.48 (5.97)	95.09 (5.26)	> 0.1

### Identification of non-synonymous variants within the katG and inhA genes in INH resistant isolates

The INH resistance of MTB isolates was frequently associated with a mutant *katG* gene, while *inhA* mutations were identified in a small number of INH resistant strains [8]. Results of custom alignment with the sequenced results using the EPI2ME desktop agent algorithm (ONT) showed more than 95% with over 200 × coverage to virtually cover the complete MTB genome (Fig. 1A), or over 100 × coverage to virtually cover the entire *katG* or *inhA* gene (Fig. 1B). No low coverage for a specific region within the MTB genome, of the *katG*, or *inhA* gene was noted.

After depleting the synonymous single nucleotide polymorphism (SNPs) found in all isolates enrolled in this study, five non-synonymous variants within the *katG* gene were identified in 10 susceptible isolates only (Table 3, upper; susceptible only). Seventeen novel variants (Table 3, upper; susceptible and resistant) and four previously-reported variants (Table 3, upper, underlined) were characterized in susceptible or INH resistant isolates. Among the variants, the minimal confidence Arg463Leu (R463L) as previously reported was identified in 40% of susceptible isolates and 52.38% of all INH resistant isolates [9]. In addition, 55 amino acid substitutions were identified in INH resistant isolates with the long-reads sequencing results. Among the identified variants, 12 novel amino acid substitutions were identified in low-level INH resistant isolates (Table 3, lower). Eleven novel variants and four previously-reported variants (Table 3, lower, underlined) were only characterized in the high-level INH resistant isolates. Twenty-four novel and four previously-identified variants (Table 3, lower, low and high INH resistant, underlined) were identified in all INH resistant isolates. Among these identified candidates, the high-confidence Ser315Thr (S315T) was only characterized in 13 out of 22 high-level INH resistant isolates (68.18%; Table 3, lower) [10].

**Table 3:** List of identified variants within *katG* gene using long-read sequencing approach in susceptible or INH-resistance MTB isolates

	Variants in katG	Isolate no.	Variant no.
Susceptible only	Leu101Arg/Ile462Asn/Ala478Val/Pro566Thr/Leu631Pro	10	5
Susceptible and Resistant	Gln50Lys/Asn51Thr/Glu81Gln/Met84Thr/Pro92Ala/Gly123Arg/Lys158Asn/Tyr304Asp/Gly307Glu/Asp311Asn/Pro325Arg/Lys327Asn/Lys356Met/Tyr390Asp/Glu399Lys/Pro443His/Arg463Leu/Leu546Pro/Thr564Pro/Leu611Pro/Val659Leu	52	21

	Variants (identified copy) in *katG*	Isolate no.	Variant no.
Low INH resistant isolate	Gly14Arg(2)/ Gly124Ala(2)/ Leu141Phe(2)/ Leu141Met(2)/ Val68Ala(2)/ Pro235Gln(2) Ser374Phe(2)/ Asp509Gly(3)/ Gly570Ala(2)/ Gly665Val(2)/ Leu704Val(2)/ Phe737Leu(2)	20	12
High INH resistant isolate	Tyr95Phe(2)/ Pro136Ala(2)/ Ser160Ala(2)/ Val188Leu(5)/ Glu287Asp(2)/ Ser315Thr(13)/ Ser315Gly(1)/ Ser315Asn(1)/ Thr354Ile(2)/ Ser376Met(3)/Gly560Val(2)/Thr579Asn(2)/Lys600Glu/Arg(2)/ Asn602Lys/Asp(3)/ Met664Leu/Ile(2)	22	15

The value highlighted in underline text presents the number of isolate harboring the amino acid substitution that has been identified in other study

In addition, 17 variants were identified within the coding region of *inhA* gene in susceptible isolates (Table 4, upper). Eight and ten amino acid substitutions within the coding region of *inhA* gene were characterized in the low-level or high-level INH resistant strains within the coding region of *inhA* gene. Among the identified candidates, the Asn139Lys and Asp150Glu variants were frequently identified within the *inhA* gene in the low-level INH resistant isolates (Table 4, lower; at a frequency of 25% and 35%, respectively). Moreover, 21 amino acid substitutions were identified within the *inhA* gene in the all INH resistant isolates (Table 4, lower).

**Table 4:** List of identified variants within *inhA* gene using long-read sequencing approach in susceptible or INH-resistance MTB isolates

	Variants in *inhA*	Isolate no.	Variant no.
Susceptible only	Ser20Pro/Pro107Leu/Ala131Gly/Pro156Gln/Ile202Val/Gly212Cys/Ile228Phe/Thr236Ala	10	8
Susceptible and Resistant	Gly14Glu/Ser20Pro/Arg27Leu/Ala75Pro/Ala81Thr/Ser94Trp/Ser152Gly/Ala72Pro	52	9

	Variants (identified copy) in *inhA*	Isolate no.	Variant no.
Low INH resistant isolate	His24Leu(2)/ Ala58Gly(2)/ Gly83Glu(2)/ Gly102Arg(2)/ Asn139Lys(5)/ Asp150Glu(7) Met232Leu(2)	20	8
High INH resistant isolate	Lys8Gln(2)/ Gly83Arg(2)/ Ala72Asp(2)/ Phe97Ser(2)/ Lys132*(3)/ Pro136Arg(2)/ Pro151Leu(2) Pro151Arg(2)/ Ser166Asn(2)/ Cys243Arg(2)	22	10
Low and High INH resistant isolate	Arg27Pro(2)/ Ala29Gly(2)/ Glu31Asp(2)/ Gly40Trp(4)/ Phe41Leu(4)/ Arg43Trp(4)/ Gln66Pro(4)Lys87Met(2)/ Lys132Glu(3)/ Lys132Gln(2)/ Pro140His(6)/ Leu168Phe(4)/ Glu219Gln(8)/ Glu219Asp(2) Asp223Asn(3)/ Trp2308(3)/ Thr254Pro(3)/ Gly262Asp(8)/ His265Tyr(4)/ Gln267His(7)/ Leu268Trp(5)	42	21

### Correlations of identified variants of the katG gene with results of the minimal inhibitory concentration test

Correlation of the Ser315Thr variant within the *katG* gene with the INH resistant signature of clinical MTB isolates was predominantly characterized worldwide [10]. In total, 10 genotyping variants within the *katG* gene, including Ser315Thr, were identified in the high-level INH resistant isolates (*n* = 20) according to the results of DST assay. The minimal inhibitory concentration (MIC) test was subsequently conducted to evaluate the impact of individual genotyping variant on the resistance signature of recruited MTB isolates toward INH. MIC results showed significant INH-resistance (2 μg/mL) with sole presence of the Ser315Thr variant within the *katG* gene (Table 5, isolates 1 to 3). The existence of Thr579Asn or Lys600Glu coupled with Ser315Thr resulted in high INH-resistance (≥ 4 μg/mL) compared to those isolates containing Ser315Thr only (Table 5, isolate 4–6). It was intriguing that high-level INH resistant (≥ 4 μg/mL) was noted in isolates only containing Thr579Asn (Table 5, isolate 7). Even though the differential results between DST and MIC assay was identified in the clinical isolates without Ser315Thr, Thr579Asn, or Lys600Glu variant (Table 5, isolate 9 and 10), the low-level INH resistance of these isolates were consistently identified by using phenotypic or genotypic analysis. Taken together, the confidence of Thr579Asn or Lys600Glu variant on the resistant signature of MTB toward INH treatment was worthy of further validation.

**Table 5:** The relevance between the identified variant within the *katG* gene and the results of DST or MIC testing

Genotyping no.	High confidence variant	Novel variant	MIC of INH (mg/mL)	DST of INH (mg/mL)	Frequency (%)
1	Ser315Thr	N/A	2	1	25% (5/20)
2	Ser315Thr	Thr376Met Pro136Ala	2	1	10% (2/20)
3	Ser315Thr	Val188Leu Ser160Ala	2	1	15% (3/20)
4	Ser315Thr	Val188Leu Thr579Asn	4	1	10% (2/20)
5	Ser315Thr	Thr579Asn	> 4	1	5% (1/20)
6	Ser315Thr	Lys600Glu	> 4	1	10% (2/20)
7	N/A	Glu287Asp Lys310Thr Thr579Asn	> 4	1	10% (2/20)
8	N/A	Glu318Gln Ile552Arg Gly599Arg	0.5	> 0.2	5% (1/20)
9	N/A	Glu287Asp Thr376Met Lys600Arg Ser315Asn	0.03	> 0.2	5% (1/20)
10	N/A	Met664Ile Ser315Gly	0.06	> 0.2	5% (1/20)

### Predictive values of identified variant toward INH resistance is estimated using receiver operating characteristic analysis

**Fig. 2:**
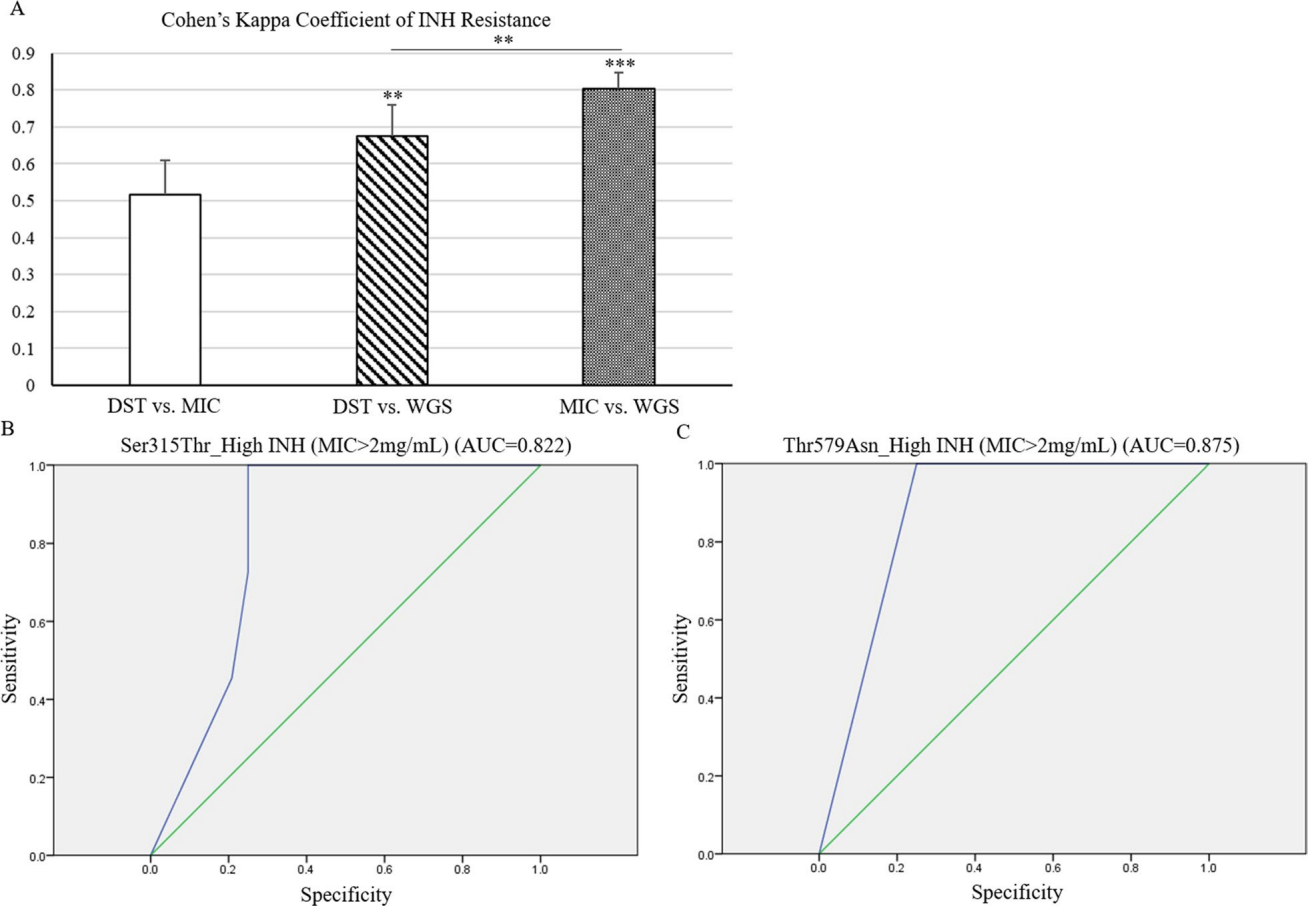
The utility of WGS sequencing on prediction of the INH-resistant signature of MTB isolates is estimated using statistical analyses. **A** The agreement between phenotypic and genotypic assay toward the INH-resistant signature of MTB was evaluated with the Cohen's Kappa index. The utility of presence of (**B**) Ser315Thr or (**C**) Thr579Asn for predicting the high INH-resistant signature of MTB was evaluated using the ROC analysis

Overall agreement between the phenotypic and genotypic approaches for INH-resistance of MTB isolates was estimated in this study. Results of WGS and MIC tests showed better agreement (Fig. 2A, dotted bar, Cohen's kappa coefficient = 0.803) than those between DST and MIC results (Fig. 2A, open bar, Cohen's kappa coefficient = 0.516) or DST and WGS results (Fig. 2A, slashed bar, Cohen's Kappa coefficient = 0.675). The predictive utility of the existence of Ser315Thr or Thr579Asn toward INH resistance of clinical isolates was estimated by using the Receiver Operating Characteristic (ROC) curve analysis. The area under ROC curve (AUC) indicated the predictive efficacy of Ser315Thr (Fig. 2B, AUC = 0.822) or Thr579Asn (Fig. 2C, AUC = 0.875) in differentiating high-level INH resistant (> 2 μg/mL) of MTB isolates recruited in this study.

## Discussion

The turnaround time of DSTs for MTB is a major challenge relevant to the efficacy of precise treatment [11]. Phenotypic methods take up to several weeks due to the low growth rate of MTB, which determines the therapeutic strategy toward MTB disease [12]. Advances in high-throughput sequencing approaches have expanded the practicality of genome analyses in clinical examinations which reduce the turnaround time of DSTs for MTB [13]. Among widely applied platforms, the ONT platform is suitable for sequencing the GC-rich or repetitive proline-glutamate regions within the MTB genome, which might be omitted in results of short-read sequencing [14]. Moreover, the increased accuracy of the ONT platform of assembling a precise MTB genome is achieved with a higher reading depth compared to that of the Illumina platform [7, 15]. The MinION quality score does not equate to the same Phred score, a mean quality score of 20.07 regarding the per base quality score of MinION sequencing were assessed using the CLC genomics workbench in this study. In addition to well-characterized variants, emerging non-synonymous variants within the MTB *katG* or *inhA* gene were identified using the ONT platform in this study. The putative impacts of these variants on the INH-resistance signature should be further demonstrated.

Ser315Thr has been widely demonstrated to be a high-confidence and frequent polymorphism that confers an INH-resistance signature of MTB [16], which was consistently characterized in our study through results of ONT sequencing. It was reported that most INH-resistant isolates harboring the Ser315Thr variant had an MIC of > 5 µg/mL [17], whereas nucleotide polymorphisms within other INH-resistant isolates with high MIC values have been sparsely investigated. In this study, results of the MIC test indicated that the presence of novel amino acid polymorphisms within the C-terminal domain of KatG conferred a high INH-resistance phenomenon. INH-resistant isolates harboring the Thr579Asn variant consistently had the highest MIC of > 4 µg/mL, which was not previously disclosed in other studies. More functional and structural analysis should be conducted to pursue the biological impacts of the Thr579Asn variant on the KatG enzyme.

Agreement between the results of the phenotypic test and genotyping analysis is considered a critical issue when evaluating the utility of WGS results on determining the drug-resistance signature of MTB infection [18]. Phenotypic drug-resistance of MTB isolates with no resistance-related variants identified using a genotypic assay is the majority of discordances [19]. The discordance could be emphasized as follows. First, the accurate coverage or assembly of a high-quality entire MTB genome using an appropriate sequencing platform, including long read-sequencing strategy, might diminish the discordance between genotyping and phenotypic results [20]. Second, sequencing results of HMW gDNA extracted from an original sputum sample or subcultured isolate is worth further comparison and optimization [21, 22]. Third, the DST analysis with MGIT 960 was demonstrated to exert incidences of false-positive drug resistance [23]. Fourth, standardization of the analytic workflow or threshold employed for the naming of nucleotide polymorphisms with sequencing results is required to acquire consistent results [24].

## Conclusions

In our study, the long read-sequencing platform was subjected to SNP calling within the entire MTB genome. In addition to the high-confidence and frequent Ser315Thr mutations, the emerging Thr579Asn variant relevant to high INH-resistance was characterized using MIC and WGS analyses. Moreover, Cohen's kappa value indicated better agreement between the MIC and WGS analyses of differentiating high INH-resistance of clinical MTB. These results suggested the practicable adoption of the ONT sequencing approach as an alternative to the current diagnostic method for DSTs of MTB.

## Data Availability

Data sharing is not applicable to this article as no datasets were generated throughout the study.

## References

[1] World Health Organization Global tuberculosis report 2018 World Health Organization.

[2] Unissa AN, Subbian S, Hanna LE, Selvakumar N (2018). Overview on mechanisms of isoniazid action and resistance in *Mycobacterium tuberculosis*. Infect Genet and Evol..

[3] Jena L, Waghmare P, Kashikar S, Kumar S, Harinath BC (2014). Computational approach to understanding the mechanism of action of isoniazid, an anti-TB drug. Int J Mycobacteriol.

[4] Li Y, Cao X, Li S, Wang H, Wei J, Liu P (2016). Characterization of *Mycobacterium tuberculosis* isolates from Hebei, China: genotypes and drug susceptibility phenotypes. BMC Infect Dis.

[5] Chang Y, Kim S, Kim Y, Ei PW, Hwang D, Lee J (2020). Evaluation of the QuantaMatrix multiplexed assay platform for molecular diagnosis of multidrug- and extensively drug-resistant tuberculosis using clinical strains isolated in Myanmar. Ann Lab Med.

[6] Xie YL, Chakravorty S, Armstrong DT, Hall SL, Via LE, Song T (2017). Evaluation of a rapid molecular drug-susceptibility test for tuberculosis. N Engl J Med.

[7] Smith C, Halse TA, Shea J, Modestil H, Fowler RC, Musser KA (2020). Assessing nanopore sequencing for clinical diagnostics: a comparison of next-generation sequencing (NGS) methods for *Mycobacterium tuberculosis*. J Clin Microbiol.

[8] Suthum K, Samosornsuk W, Samosornsuk S (2020). Characterization of katG, inhA, rpoB and pncA in *Mycobacterium tuberculosis* isolates from MDR-TB risk patients in Thailand. J Infect Dev Ctries.

[9] Purkan P, Ihsanawati I, Natalia D, Syah YM, Retnoningrum DS, Siswanto I (2018). Molecular analysis of katG encoding catalase-peroxidase from clinical isolate of isoniazid-resistant *Mycobacterium tuberculosis*. J Med Life.

[10] Charoenpak R, Santimaleeworagun W, Suwanpimolkul G, Manosuthi W, Kongsanan P, Petsong S (2020). Association between the phenotype and genotype of isoniazid resistance among *Mycobacterium tuberculosis* isolates in Thailand. Infect Drug Resist.

[11] Ruesen C, Riza AL, Florescu A, Chaidir L, Editoiu C, Aalders N (2018). Linking minimum inhibitory concentrations to whole genome sequence-predicted drug resistance in *Mycobacterium tuberculosis* strains from Romania. Sci Rep.

[12] Kwak N, Choi SM, Lee J, Park YS, Lee CH, Lee SM (2013). Diagnostic accuracy and turnaround time of the Xpert MTB/RIF assay in routine clinical practice. PLoS ONE.

[13] Li MC, Chen R, Lin SQ, Lu Y, Liu HC, Li GL (2020). Detecting ethambutol resistance in *Mycobacterium tuberculosis* Isolates in China: a comparison between phenotypic drug susceptibility testing methods and DNA sequencing of embAB. Front Microbiol.

[14] Chao Y, Li J, Gong Z, Li C, Ye M, Hong Q (2021). Rapid discrimination between tuberculosis and sarcoidosis using next-generation sequencing. Int J Infect Dis.

[15] Chan WS, Au CH, Chung Y, Leung HCM, Ho DN, Wong EYL (2020). Rapid and economical drug resistance profiling with Nanopore MinION for clinical specimens with low bacillary burden of *Mycobacterium tuberculosis*. BMC Res Notes.

[16] Dippenaar A, Goossens SN, Grobbelaar M, Oostvogels S, Cuypers B, Laukens K (2021). Nanopore sequencing for *Mycobacterium tuberculosis*: a critical review of the literature, new developments and future opportunities. J Clin Microbiol.

[17] de Vos M, Scott L, David A, Trollip A, Hoffmann H, Georghiou S (2021). Comparative analytical evaluation of four centralized platforms for the detection of *Mycobacterium tuberculosis* complex and resistance to rifampicin and isoniazid. J Clin Microbiol.

[18] Guo H, Seet Q, Denkin S, Parsons L, Zhang Y (2006). Molecular characterization of isoniazid-resistant clinical isolates of *Mycobacterium tuberculosis* from the USA. J Med Microbiol.

[19] Banu S, Rahman SM, Khan MS, Ferdous SS, Ahmed S, Gratz J (2014). Discordance across several methods for drug susceptibility testing of drug-resistant *Mycobacterium tuberculosis* isolates in a single laboratory. J Clin Microbiol.

[20] Tyler AD, Christianson S, Knox NC, Mabon P, Wolfe J, Van Domselaar G (2016). Comparison of sample preparation methods used for the next-generation sequencing of *Mycobacterium tuberculosis*. PLoS ONE.

[21] Doyle RM, Burgess C, Williams R, Gorton R, Booth H, Brown J (2018). Direct whole-genome sequencing of sputum accurately identifies drug-resistant *Mycobacterium tuberculosis* faster than MGIT culture sequencing. J Clin Microbiol.

[22] Mokaddas E, Ahmad S, Eldeen HS, Al-Mutairi N (2015). Discordance between Xpert MTB/RIF assay and Bactec MGIT 960 culture system for detection of rifampin-resistant *Mycobacterium tuberculosis* isolates in a country with a low tuberculosis (TB) incidence. J Clin Microbiol.

[23] Cayci YT, Bilgin K, Coban AY, Birinci A, Durupınar B (2017). An evaluation of false-positive rifampicin resistance on the Xpert MTB/RIF. Mem Inst Oswaldo Cruz.

[24] Zhang C, Xiu L, Li Y, Sun L, Li Y, Zeng Y, Wang F, Peng J (2021). Multiplex PCR and nanopore sequencing of genes associated with antimicrobial resistance in Neisseria gonorrhoeae directly from clinical samples. Clin Chem.

